# Count Your Eggs Before They Invade: Identifying and Quantifying Egg Clutches of Two Invasive Apple Snail Species (*Pomacea*)

**DOI:** 10.1371/journal.pone.0077736

**Published:** 2013-10-16

**Authors:** Colin H. Kyle, Allyson L. Plantz, Therese Shelton, Romi L. Burks

**Affiliations:** 1 Department of Ecology and Evolution, University of Chicago, Chicago, Illinois, United States of America; 2 Department of Biology, Southwestern University, Georgetown, Texas, United States of America; 3 Department of Mathematics and Computer Science, Southwestern University, Georgetown, Texas, United States of America; Gettysburg College, United States of America

## Abstract

Winning the war against invasive species requires early detection of invasions. Compared to terrestrial invaders, aquatic species often thrive undetected under water and do not garner notice until too late for early action. However, fortunately for managers, apple snails (Family Ampullariidae, Genus *Pomacea*) provide their own conspicuous sign of invasion in the form of vibrantly colored egg clutches. Managers can potentially use egg clutches laid in the riparian zone as a means of early detection and species identification. To facilitate such efforts, we quantified differences in characteristics (length, width, depth, mass, egg number) of field-laid clutches for the two most common invasive species of apple snail, *P. canaliculata* and *P. maculata*, in native and non-native populations. *Pomacea canaliculata* native and non-native populations differed noticeably only in width. Native *P. maculata* clutches possessed significantly greater width, mass and eggs numbers compared with native *P. canaliculata*. Non-native *P. maculata* clutches significantly exceeded all other populations in all measured characteristics. Consequently, these traits may successfully distinguish between species. Fecundity data also allowed us to develop models that accurately estimated the number of eggs per clutch for each species based on clutch dimensions. We tested one, two and three dimensional models of clutches, including rendering a clutch as either a complete ellipsoid or an ellipsoid intersected by a cylinder to represent the oviposition site. Model comparisons found the product of length and depth, with a different function for each population, best predicted egg number for both species. Comparisons of egg number to clutch volume and mass implied non-native *P. canaliculata* may be food limited, while non-native *P. maculata* appeared to produce such enormous clutches by having access to greater nutrients than the native population. With these new tools, researchers and managers can quickly identify, quantify and begin eradication of new non-native apple snail populations.

## Introduction

Most invasions of non-native species fail [1]. However, the select few with the biological adaptations necessary to succeed in novel environments clearly defy the odds while simultaneously reinforcing the need for quick action by managers [2]. Consequently, early detection of non-native, invasive species (NIS) holds the best potential to avoid the considerable economic and ecological costs that often result from successful establishment [1,3–5]. Applications of molecular biology now use environmental DNA (eDNA) to test for the presence of unwanted NIS [6-8]. While these methods prove successful for several species, the procedures take considerable time and expertise to develop and remain costly to execute. Ultimately, such an investment will pay off for rare or cryptic species that leave few other signs of invasion [7].

On the other hand, routine field observations and a basic knowledge of natural history may prove the best means of detecting early invasions, especially for those aquatic species that leave signs of their presence in the riparian habitat. For example, red swamp crayfish, *Procambarus clarkii*, modify shorelines as a result of burrowing for shelter [9]. In addition, bullfrogs (*Lithobates catesbeianus*) deposit visibly large masses of eggs at the base of vegetation [10]. More often than not, managers might stumble upon these signs of invasions considerably before they observe an actual adult invader.

**Figure 1:**
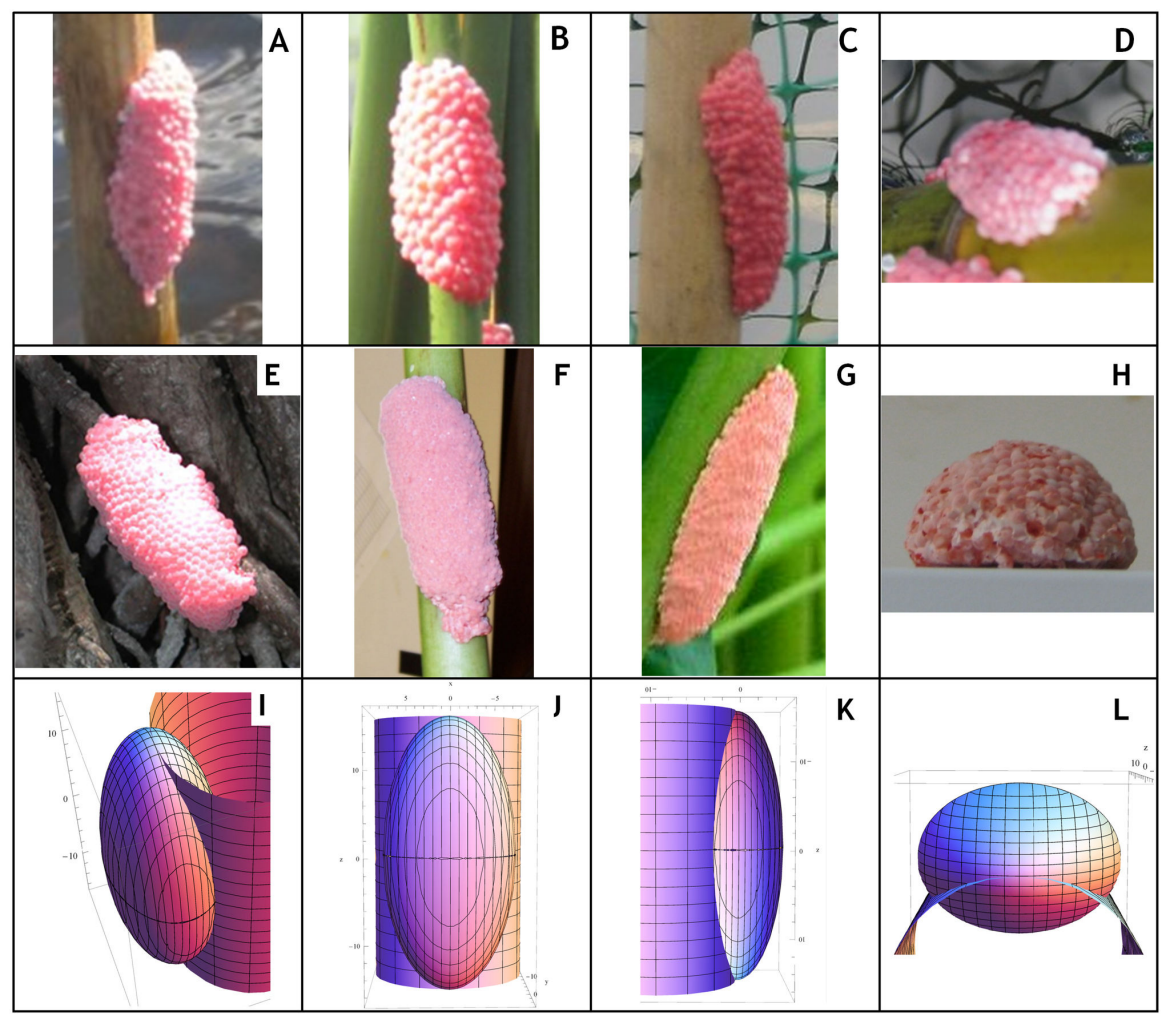
Photographs of apple snail egg clutches and renderings of Intersected Model at different angles. Top row (A-D) contains *Pomacea canaliculata* clutches and the middle row (E-H) contains *Pomacea maculata* clutches. The bottom row (I-L) displays renderings in Mathematica 7.01 of the Intersected Model we attempted to use to estimate the volume of apple snail egg clutches.

South American apple snails (genus *Pomacea*) represent a recently acknowledged, highly destructive and quickly spreading group of aquatic invaders [11,12]. With some species reaching fist size or larger (shell height and operculum width up to 55 mm), competing with native species [13], feeding on live plant tissue [14-17] and possessing high fecundity [18], apple snails can completely strip water bodies of aquatic and emergent vegetation [19]. Furthermore, apple snails possess shells with a generic round shape and muddy brown coloration. Consequently, adults typically remain submerged and slip past managers by frequently burrowing in mud [12]. However, fortunate for managers, apple snails also possess highly visible egg masses. To reproduce, adult females of the two most common invasive species (*P. canaliculata* and *P. maculata*) climb up stems of riparian vegetation, or other emergent structures such as concrete walls, wooden docks or cisterns, to deposit vibrant pink egg clutches (Figure 1a-h) above the water line [16,20]. The conspicuousness and general accessibility of these egg clutches gives us an opportunity to develop tools that will help managers and researchers find and effectively respond to apple snail invasions as early and efficiently as possible [2].

Apple snail export from South America began when people in Southeast Asia imported the snails in the 1980s as a potential human food source that unintentionally resulted in devastating losses to rice production when the snails escaped captivity [12]. Accidental introductions to North America, Europe and the Pacific Islands [21,22] quickly followed. Collectively, humans have most frequently exported two species of apple snail, *P. canaliculata* (Lamarck 1828) and *P. maculata*, formerly *P. insularum* (d’Orbigny 1835) [23]. Analogous morphological characteristics and similar reproductive strategies have often resulted in the misidentification of *P. canaliculata* and *P. maculata* as the same species or mistakenly as sister species in literature [23]. Furthermore, laypersons and land managers alike frequently misidentify the prominent egg clutches, either as the incorrect snail species or as frog or insect eggs (Kyle and Burks, personal observations). For example, along the Gulf Coast of the United States, recently discovered populations of *P. maculata* quickly outpaced management efforts, especially in Florida and Alabama [11,24,25]. Misidentification of the apple snails as *P. canaliculata* [23] probably resulted in an underestimation of the reproductive potential as *P. canaliculata* only reproduces at roughly a quarter of the rate of the actual invader *P. maculata* [18,24,26]. 

Because of the taxonomic confusion, ecologists must now rely primarily on genetic verification to classify accurately these organisms [23]. Unfortunately, not every researcher or land manager has access to genetic analysis. However, each apple snail species tends to produce egg clutches with distinguishable features and dimensions [12,23]. While descriptions of individual species can be found [18,20], to our knowledge, no quantitative assessment of the differences in egg clutches between different species or in native versus non-native habitats currently exists for apple snails. Determination of distinguishable trends in clutch characteristics potentially could enable identification of the nuisance species through simple measurements and counts of easily recognizable egg clutches.

While apple snail eggs are easy to find, clutches can contain from several dozen up to almost five thousand individual eggs [8,12] and manual counting eggs in a single clutch requires substantial time. Furthermore, researchers recently found that female apple snails produce egg clutches at a consistent rate throughout the breeding season and across a range of resource availabilities but egg number within clutches varied. Female snails with access to unlimited food produced over three times the number of eggs per clutch and total eggs per season compared to food-limited treatments [27]. Because of potential range in clutch sizes, surveys that only count egg clutch numbers and do not quantify individual egg densities could easily misidentify the invasive potential of non-native populations. 

To capitalize on the unique reproductive strategy of apple snails, we first quantified differences in the characteristics (length, width, depth, mass, egg number) of field-oviposited clutches for *P. canaliculata* and *P. maculata* in native (Uruguay) and non-native populations (Hawaii and Texas, USA, respectively). We also utilized our fecundity data to develop models that best estimated the number of eggs per clutch and clutch volume for each species using measured clutch dimensions (length, width, depth). We avoided using mass to predict egg number because a study documenting *P. maculata* fecundity [18] previously found mass varied widely and inconsistently predicted the number of eggs per clutch across the reproductive season, in part due to changes in mass as clutches dry out during egg development. Individual egg size, conversely, remained consistent within species, between clutches, over time [8,27], and across a range of food availability [27], indicating the physical volume and dimensions represent more reliable and broadly applicable measures on which to base estimations of egg number. Collection of additional field data allowed us to then test the utility of this model on another native apple snail species (*P. megastoma*) that has yet to surface as a global invasive species. If we can identify consistent trends in egg clutch characteristics, we may be able to give researchers and managers tools to identify apple snail species based on readily observable egg clutches alone and then more quickly quantify the reproductive potential of the invading population [2,5,26].

## Materials and Methods

### Data Collection

Researchers collected all egg clutches in this study from wild field populations of two apple snail species, *P. maculata* and *P. canaliculata* (Table 1). In the United States, we worked under United States Department of Agriculture Plant Protection and Quarantine guidelines (Permit P-526-100106-007) and permission from Texas Parks and Wildlife (Exotic Species Permit 03-04-074). To test the applications of our methods beyond these two species, we also collected *P. megastoma* clutches (n=43) from native populations along the west coast of Uruguay. Work in Uruguay did not require permits for the described study, which complied with all relevant regulations and did not involve any protected or endangered species. To ensure accurate identification of clutches, we specifically collected all eggs from snail populations who’s species had recently been confirmed using genetic and anatomic analyses (see Table 1 in 23] and compared clutch characteristics with previously published accounts for each species where available [13,18,21]. 

**Table 1:** Descriptive information about apple snail egg clutches.

**Species**	*P. canaliculata*		*P. maculata*		Normality Test (p-value)	F or H value	P value
**Population**	Native	Non-Native	Native	Non-Native			
**Location**	Eastern Coast of Uruguay	Hawaii	Western Coast of Uruguay	Houston, Texas			
**N**	42	97	27	72			
**Length (mm)**	30.3± 2.11^ab^	27.7± 0.764^a^	42.3± 4.06^b^	58.4± 1.57^c^	< 0.0001	H = 116.7	< 0.0001
**Width (mm)**	14.67± 1.57^a^	17.6±0.265^b^	18.6±0.750^b^	22.9±0.370^c^	0.302	F= 67.1	< 0.0001
**Depth (mm)**	9.07±0.362^a^	10.1±0.273^a^	10.2±0.413^a^	18.2±0.343^c^	< 0.0001	H = 144.7	< 0.0001
**Ellipsoid Volume (mm^3^)**	2502.1±318.6^a^	2652.2±124.5^a^	4597.0±577.7^a^	12816.7±498.0^b^	< 0.0001	H =149.7	< 0.0001
**Mass (g)**	2.34±0.279^a^	1.71±0.0747^a^	5.74±0.865^b^	8.55±0.361^c^	< 0.0001	H =119.1	< 0.0001
**Egg Number per Clutch**	192.0±27.0^a^	147.1±6.06^a^	867.6±143.4^b^	2028.6±77.0^c^	< 0.0001	H =149.9	< 0.0001

Mean clutch characteristics (± one standard error) and statistical test results for each apple snail population we studied. Lower case letters denote statistically significant differences. Tests comparing apple snail populations each had 3 degrees of freedom.

Researchers searched water-side, emergent vegetation and objects for egg clutches. Due to the highly heterogeneous environment in Uruguay, the sample of native *P. canaliculata* and *P. maculata* included clutches oviposited on a range of plant species and wooden poles. In Hawaii, we collected non-native *P. canaliculata* clutches off of *Pistia stratiotes* (water lettuce) and a *Typha* (cattail) species. The non-native *P. maculata* population in Texas, however, tended to oviposit disproportionately on wild taro plants (*Colocasia esculenta*), and we collected clutches only on this plant species [16,20]. *Pomacea megastoma* clutches occurred primarily on flat, hard rock surfaces (Burks, unpublished data). Apple snail egg clutches also frequently occur on emergent man-made structures, such as bridges, docks and boats [20]. We focused largely on natural substrates in our surveys because apple snails disproportionately oviposit on woody and herbaceous structures rather than artificial structures when available in the lab [16] and the field [20]. 

Removal of clutches from their substrates with razor blades minimized damage and allowed collection of primarily intact, unhatched clutches. After removal from substrate, we measured clutch dimensions (length, width, depth) with digital calipers (± 0.01 mm) and weighed each clutch to the nearest tenth of a gram on a field scale. We determined the number of individual eggs per clutch (EPC) by dissolving the gelatinous matrix of each clutch in a 10% NaOH solution and then manually counting the separated eggs [18].

### Clutch Differences between Populations

With four independent populations of apple snails (Table 1), we explored whether clutch characteristics (length, width, depth, mass, ECP, and estimated volume using the Ellipsoid Model described below) differed between species and if they varied significantly between native and non-native habitats. Because the native and non-native populations we tested occurred in distinct regions, we did not group data by species or residence (i.e. native vs. non-native) and treated all four populations as independent for statistical purposes. We first compared the distribution of measurements for each characteristic to a normal distribution using Shapiro-Wilk normality tests [28]. All attempted transformations to non-normal data failed to restore normality. In the one case (i.e. clutch width) where the data met a normal distribution (p>0.05), we tested for differences between populations using a one-way ANOVA followed by a Tukey’s Post-Hoc Test [29]. We utilized Kruskal-Wallis tests [29] followed by a Multiple Comparison Between Treatments Test [30] to compare snail populations when data for a characteristic failed to resemble a normal distribution. We used the statistical software R to conduct our analyses [28]. 

### Predicting Number of Eggs per Clutch

To enable future researchers to estimate EPC without manually counting every egg, we investigated the predictive ability of readily measured clutch dimensions (length, width, depth) for each species. Because EPC represent count data, we utilized Generalized Linear Models (GLMs) with Poisson distributions to estimate model coefficients for predicting manual egg counts based on selected variables [28]. We compared nested models of increasing complexity, beginning with the effect of Residence (native vs. non-native population). We then progressively added all combinations of clutch dimension products as predictive variables (see Table 2), which allowed us to compare the forecasting potential of each dimension and determine how much additional clutch measures improved the accuracy of the model [28]. We tested both additive and interactive relationships between Residence and clutch dimensions, which produced models in which each population has its own EPC predicting function. An additive relationship between Residence and dimensions resulted in EPC functions with different intercepts but the same slope. Both an additive and interactive relationship yielded each population EPC functions with different intercepts and slopes [28]. We used Akaike’s Information Criterion (AIC) to compare all nested model fits and consider a ΔAIC of greater than three points a significantly less useful model [31].

**Table 2:** Generalized Linear Model comparisons by species.

**Model**	**Number of Parameters (K)**	***P. canaliculata* ΔAIC (N=138)**	***P. maculata* ΔAIC (N=99)**
Residence	2	5442	23,276
Residence * Length	4	982	3613
Residence * Width	4	1086	12,822
Residence * Depth	4	3027	18,301
Residence * Length×Width	4	254	1193
Residence * Length×Depth	4	0	0
Residence * Width×Depth	4	1683	12150
Residence * Length×Width×Depth (Ellipsoid Model)	4	73.5	54.5
Residence * (Length×Width×Depth - V_S_) (Intersected Model)	6	97.2	

Comparisons of nested Generalized Linear Models with a Poisson distribution to predict the number of eggs contained in a clutch using clutch characteristics. The variable “Residence” distinguishes between native and non-native populations for each species. The parameter V_S_ represents the volume occupied by the substrate in the Intersected Model. The Intersected Model for *Pomacea maculata* converged on the Ellipsoid Model, so no result is given for that model. Multiplication signs (×) indicate interactive relationships between variables and asterisks (*) indicate both additive and interactive relationships between variables.

To improve our ability to estimate EPC, we investigated the utility of using clutch volume, rather than individual dimensions, to predict EPC. We investigated two different mathematical methods of estimating clutch volume based on clutch dimensions. Because egg clutches of apple snails possess a consistent, elongated rounded shape (Figure 1, top two rows), we first modeled egg clutches as complete ellipsoids (volume = lwdπ/6; further referred to as “Ellipsoid Model”). Because the Ellipsoid model only multiplies the product of the three dimensions by a constant (π/6), finding the relationship between volume predicted by the Ellipsoid model and EPC produced the same result as the above described nested model using the product of all three dimensions. 

The process of rendering, a clutch as a full ellipsoid, however, ignored the volume occupied by the substrate supporting the eggs. Accounting for presence of the substrate should produce a better estimate of EPC (Figure 1i-l). We therefore also estimated clutch volumes as ellipsoids intersected by a cylindrical oviposition site of optimized radius (further referred to as “Intersected Model”). To generate the Intersected Model, we constructed a numerical integration algorithm using R that calculated the volume of an ellipsoid with given dimensions intersected by an off-center cylinder of a specific radius. We centered the three dimensional function for the ellipsoid on the origin and set the outer edge of the cylinder function to run along the z-axis (Figure 1l). With this function, an input of the dimensions of a clutch and the estimated radius of the oviposition site yielded the approximate volume occupied by eggs only.

Because field crews did not measure the dimensions of the substrates on which clutches occurred, we estimated the optimal value for the radius of the oviposition site for each apple snail species and population by picking a value for the radius and calculating the volume of all clutches within the data set as if intersected by that size of substrate. Use of GLMs with Poisson distributions then allowed us to calculate a relationship between the estimated volume and actual EPC counts. By repeating this process with varying radius values and comparing the AIC values of the model for each radius, we found the most likely size of substrate on which females laid clutches in that population to use in our Intersected Model [28,31]. 

### Inferring Egg Characteristics Following Invasion

By evaluating the relationships between EPC and clutch estimated volume or mass, we can make inferences about the volume or mass of individual eggs within a clutch. If we examine these relationships between the native and non-native populations for each snail species, we can draw conclusions about how introduction to a non-native environment may have impacted egg characteristics. To do so, we compared the AIC values of GLMs in which volume or mass had either an additive relationship with Residence or an additive and interactive relationship with Residence. We then compared the slopes of the relationships between ECP and mass or Ellipsoid Model volume for non-native and native populations.

## Results

**Figure 2:**
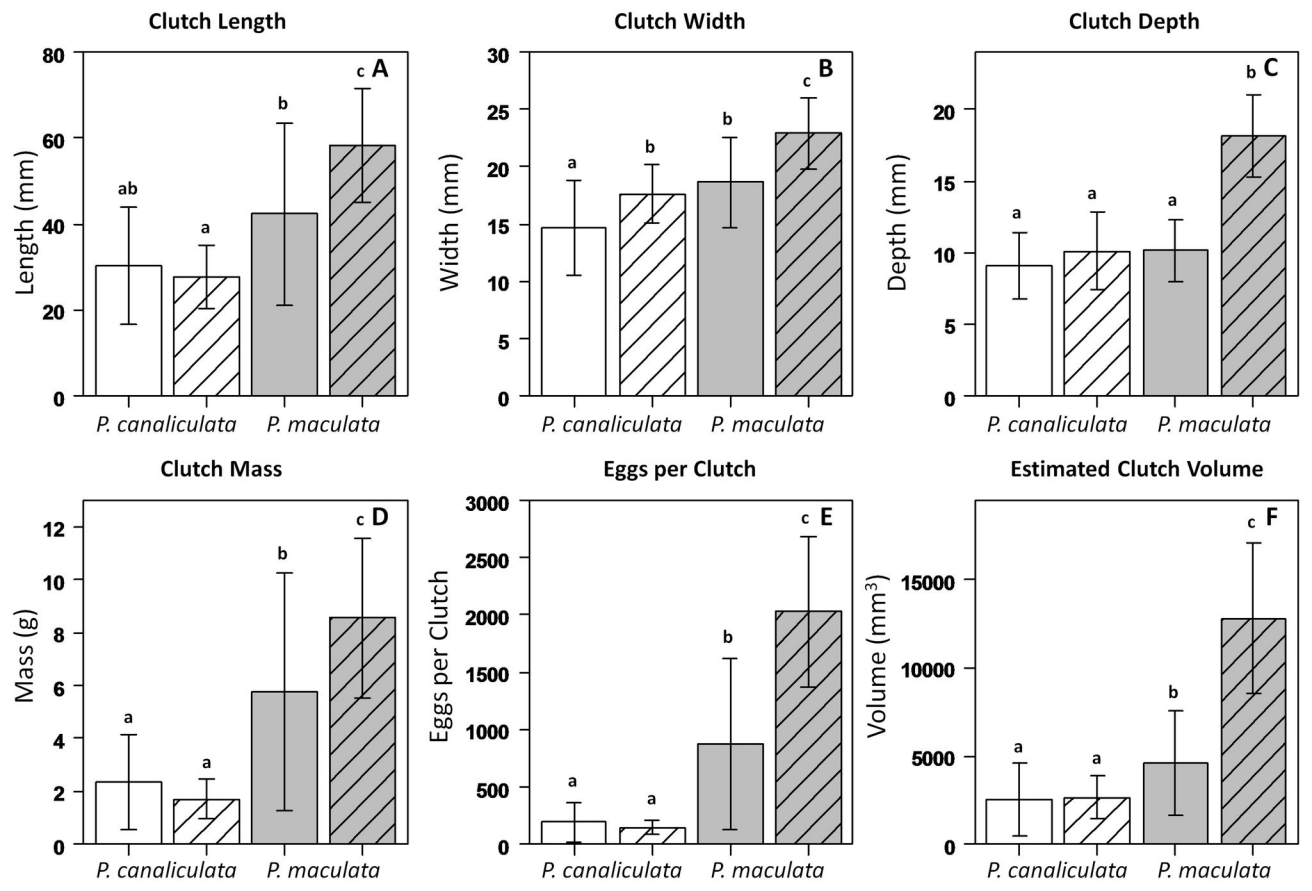
Comparison of egg clutch characteristics by population. White bars represent *Pomacea canaliculata* and grey bars represent *Pomacea maculata*. Open and hash marked bars signify data from native and non-native populations, respectively. Lower case letters denote statistically significant differences and error bars symbolize one standard deviation. We estimated clutch volume (F) using the Ellipsoid Model (length×width×depth×π/6).

After comparing the shapes and sizes of apple snail clutches, we found significant differences between the populations in all measured characteristics (Table 1, Figure 2). Only width measurements significantly resembled a normal distribution (Table 1, Column titled “Normality Test”). Clutches from *P. canaliculata* native and non-native populations only differed noticeably in width. Native *P. maculata* clutches exhibited significantly greater width, mass, EPC and estimated volume than native *P. canaliculata*. Non-native *P. maculata* clutches significantly exceeded all other populations in all measured characteristics (Figure 2).

**Figure 3:**
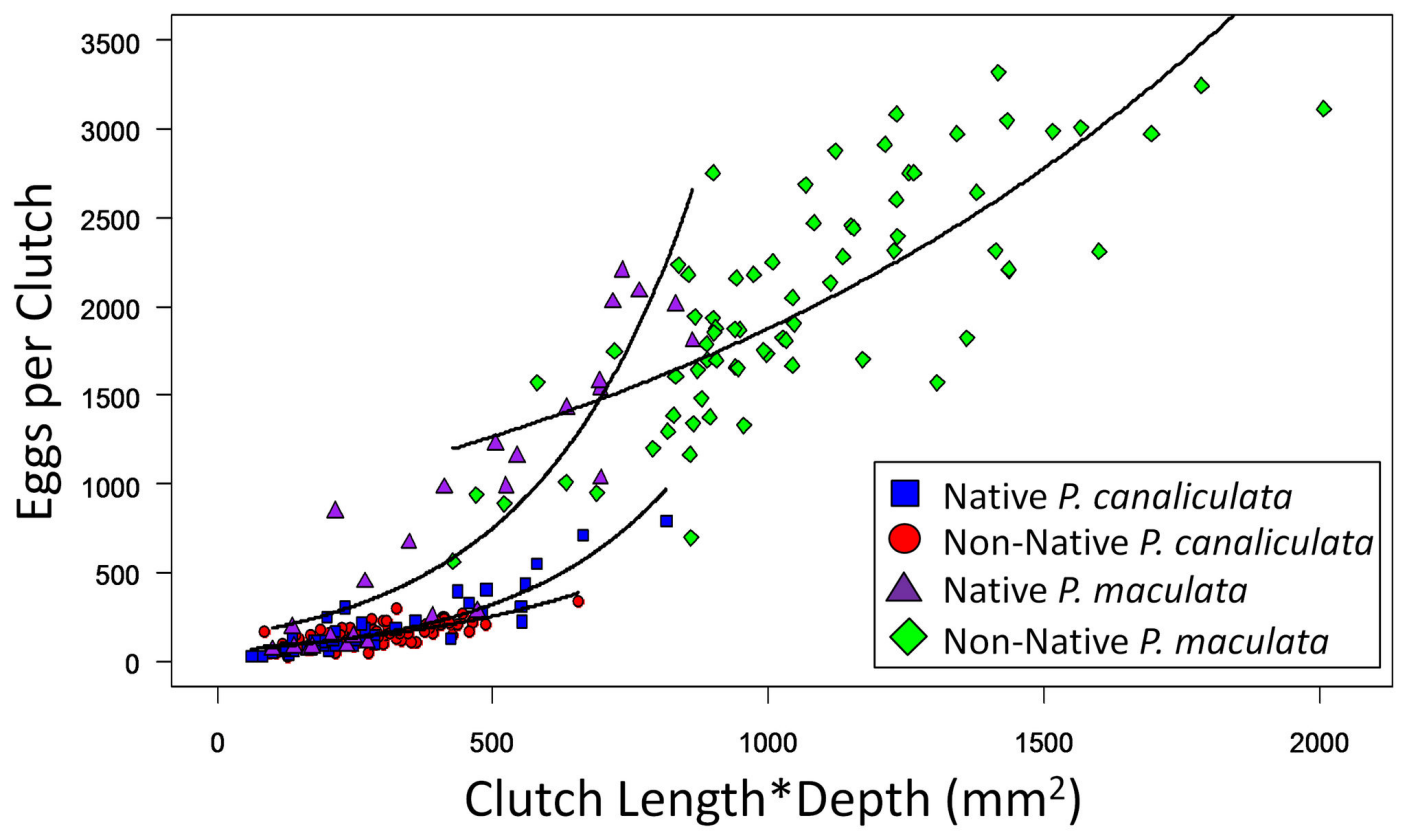
Comparison of model predictions to manual egg counts for each population. Generalized Linear Model predictions of the number of eggs per clutch using the product of clutch length and depth for each apple snail population (black lines). Coefficients for these functions can be found in Table 3.

When exploring our ability to predict EPC based on clutch dimensions, we found the product of clutch length and depth best predicted EPC for both snail species (ΔAIC > 50; Table 2; Figure 3). Utilizing all three dimensions in the model (the Ellipsoid Model) represented the next best option. Of the individual dimensions, clutch length predicted EPC significantly better than width or depth (ΔAIC > 100; Table 2). Using separate models with different slopes and intercepts for native and non-native populations always significantly improved model fits for each species (ΔAIC > 100). Therefore, we only reported models in which Residence possessed an additive and interactive relationship with the product of clutch dimensions in Table 2. 

 To improve our ability to predict EPC, we tested two models to estimate the volume of an egg clutch. For both species, the Ellipsoid Model produced greater variance and low predictive ability than a model dependent on only length and depth (Table 2). In the Intersected Ellipsoid Model, the fitting routine estimated that *P. canaliculata* clutches likely occurred on cylindrical substrates with radii of 10.4 mm and 77.0 mm in its native and non-native ranges, respectively. The program results indicated that native female *P. maculata* likely oviposited clutches on substrates with radii of 7.94 mm. However, the program failed to find a substrate size that improved the model’s predictions of non-native *P. maculata* clutches and converged on the Ellipsoid Model. While the Intersected Model incorporated biologically intuitive features, the fit did not justify the additional complexity of the model (ΔAIC = 23.7; Tables 2, 3). We utilized the Ellipsoid Model to estimate clutch volume for further statistical tests. Future references to clutch volume refer specifically to estimates from the Ellipsoid Model.

**Table 3:** Coefficients for best fitting models predicting egg number per clutch.

**Explanatory Variable**	**Population**		**Intercept**	**Slope**
Length	*P. canaliculata*	Native	4.33 ± 4.21×10^-2^ (127)	4.30×10^-2^ ± 1.20×10^-3^ (39.5)
		Non-Native	4.31 ± 3.07×10^-2^ (140)	2.41×10^-2^ ± 1.01×10^-3^ (23.8)
	*P. maculata*	Native	5.12 ± 2.17×10^-2^ (499)	3.29×10^-2^ ± 3.48×10^-4^ (135)
		Non-Native	6.65 ± 1.17×10^-2^ (570)	1.62×10^-2^ ± 1.85×10^-4^ (87.5)
Length×Depth	*P. canaliculata*	Native	4.02 ± 2.45×10^-2^ (164)	3.51×10^-3^ ± 5.09×10^-5^ (44.5)
		Non-Native	4.21 ± 2.49×10^-2^ (169)	2.67×10^-3^ ± 7.57×10^-5^ (35.2)
	*P. maculata*	Native	4.88 ± 2.11×10^-2^ (619)	3.49×10^-3^ ± 3.18×10^-5^ (179)
		Non-Native	6.75 ± 9.65×10^-3^ (700)	7.86×10^-3^ ± 8.16×10^-5^ (96.4)
Length×Width×Depth	*P. canaliculata*	Native	4.35 ± 2.85×10^-2^ (215)	1.47×10^-4^ ± 3.81×10^-6^ (44.7)
		Non-Native	4.37 ± 2.02×10^-2^ (216)	1.16×10^-4^ ± 3.18×10^-6^ (36.4)
	*P. maculata*	Native	5.11 ± 2.05×10^-2^ (742)	1.49×10^-4^ ± 1.36×10^-6^ (183)
		Non-Native	6.91 ± 8.32×10^-3^ (830)	2.78×10^-5^ ± 2.99×10^-7^ (93.1)
Mass	*P. canaliculata*	Native	4.65 ± 1.01×10^-2^ (193)	3.77×10^-1^ ± 1.15×10^-2^ (37.8)
		Non-Native	4.24 ± 2.15×10^-2^ (197)	4.10×10^-1^ ± 1.01×10^-2^ (40.7)
	*P. maculata*	Native	5.47 ± 1.74×10^-2^ (730)	1.72×10^-1^ ± 1.71×10^-3^ (149)
		Non-Native	6.87 ± 8.48×10^-3^ (810)	8.44×10^-2^ ± 8.63×10^-4^ (97.8)

Coefficients for model with lowest AIC value for each *Pomacea* species (Table 2). Coefficients include ± one standard error and the corresponding Z-Value in parentheses. The Z-value for each coefficient proved significant (z < 0.0001).

**Figure 4:**
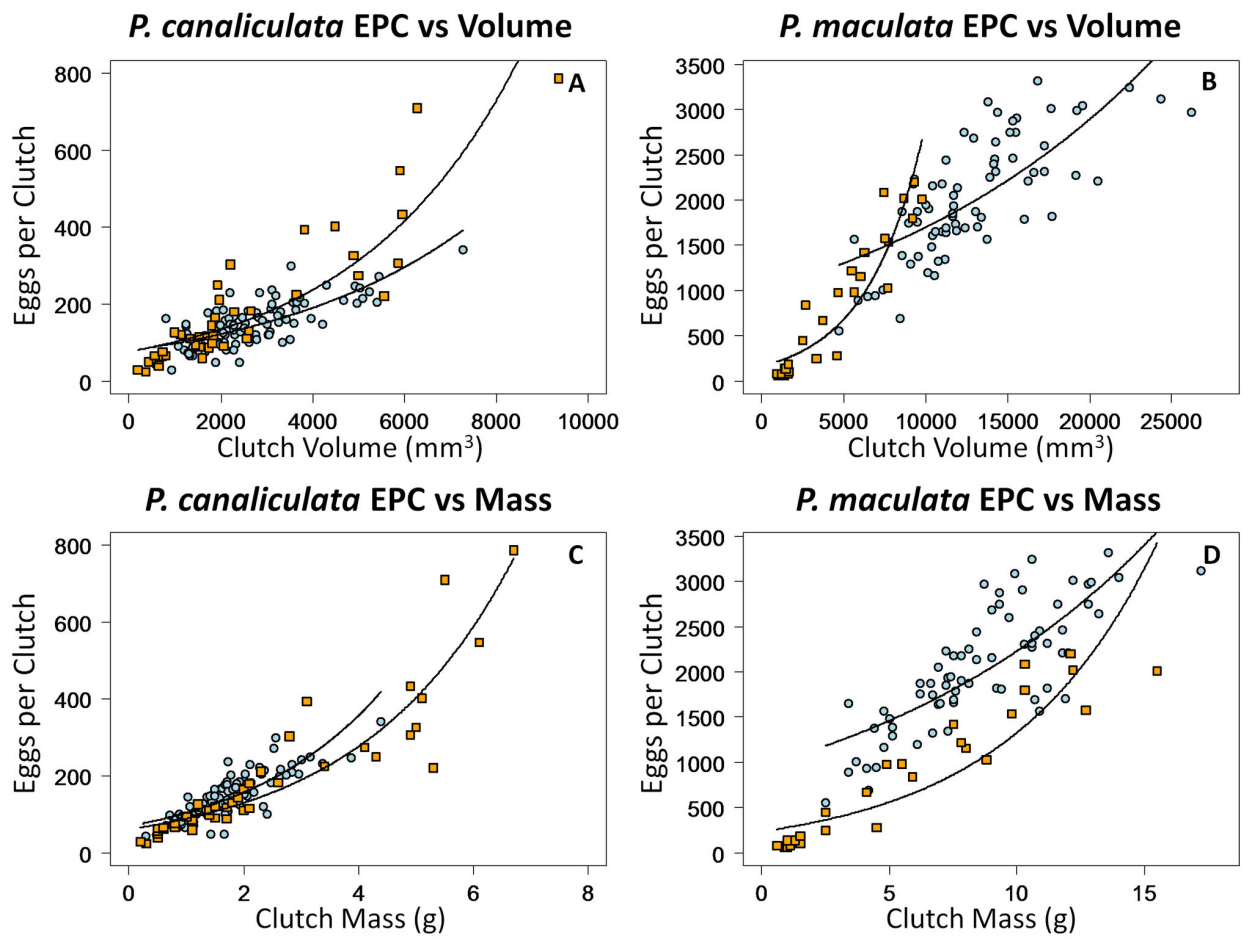
Relationships between egg number per clutch and clutch volume and mass. Results from Generalized Linear Models demonstrating the relationship between the number of eggs per clutch and estimated volume from the Ellipsoid Model (length×width×depth×π/6) (a, b) and clutch mass (c, d) for native and non-native apple snail populations. Orange filled squares represent clutches from the native population and light blue filled circles represent clutches from the non-native population. Coefficients for these functions can be found in Table 3.

We found different relationships between ECP and clutch volume or mass for native and non-native populations of both species (Table 3). We observe a significantly higher slope in the relationship between estimated clutch volume and EPC for native compared to non-native apple snail populations (*P. canaliculata* and *P. maculata*, respectively: ΔAIC = 67 and 9,180; Table 3; Figure 4a,b). Native *P. canaliculata* clutches possessed a significantly lower slope in the relationship between mass and EPC compared to the non-native population (ΔAIC = 6; Table 3; Figure 4c). For *P. maculata*, native clutches expressed a higher slope in the relationship between mass and EPC (ΔAIC = 2,690; Table 3; Figure 4d). 

When applied to a third species apple snail, the product of all three clutch dimensions most accurately predicted *P. megastoma* EPC (ΔAIC = 2.40), followed by the product of length and width (ΔAIC = 84.2). When using only a single dimension, clutch depth best predicted EPC (ΔAIC = 76.0). We can recognize *P. megastoma* clutches by their greater width (mean ± SE = 27.3mm ± 0.754; Kruskal Wallis Test: χ^2^ = 83.3, P < 0.0001), depth (mean ± SE = 16.9mm ± 0.500; Kruskal Wallis Test: χ^2^ = 92.4, P < 0.0001) and mass (mean ± SE = 8.45g ± 0.593; Kruskal Wallis Test: χ^2^ = 7.84, P < 0.0494) compared to the other two native species.

## Discussion

Based exclusively on egg clutch characteristics, we found clearly recognizable trends that researchers and managers can use to distinguish the two most common non-native invasive apple snail species. *Pomacea canaliculata* clutches exhibited smaller sizes with a lower number of larger eggs per clutch. In our study, *P. canaliculata* clutch size conservatively never exceeded 400 eggs, although a study in the native habitat documented the range of clutch size from 120 to 478 eggs in Argentina [32]. In contrast, researchers can recognize *P. maculata* clutches by their large size and much higher numbers of smaller eggs. Clutches from this species routinely contained more than a thousand eggs, with exceptionally large clutches occurring in its non-native range. Curiously, a study in Asia [33] documented up to 3000 eggs in a “*P. canaliculata*” clutch during a study of density effects on growth and reproduction of apple snails. In this case, based on recent differentiation between the two species [23], we believe the species to be misidentified as *P. canaliculata* as its reproductive characterization matches that of *P. maculata*. Thus, using these fecundity parameters and further descriptors of differences between the two species may also inform reading of past studies [23].

Comparing native and non-native populations, we found the introduction into a novel environment caused varying degrees of changes to egg clutches in each apple snail species. *Pomacea canaliculata* clutches from native and non-native populations only differed significantly in width, but native clutches possessed a wider range of sizes and reached higher EPC values. We found a higher ratio between estimated clutch volume and EPC and a lower ratio between mass and EPC for native compared to non-native clutches, implying non-native clutches contain eggs larger in size but slightly lower in mass than natives. Combined with the results from a recent experiment in which female *P. canaliculata* on reduced diets produced clutches with reduced egg numbers but largely conserved egg mass and clutch shape [27], our results indicate the non-native apple snail population in Hawaii may be in a food-limited state. In addition, the dominance of *Pistia* in the non-native habitat may have constrained clutch morphology, but the overall similarity in *P. canaliculata* clutches will make clutches comparable for species identification for newly invaded habitats.

Most strikingly, we found dramatic differences between native and non-native *P. maculata* egg clutches. Along the US Gulf Coast, *P. maculata* females produced clutches larger in all measured characteristics and containing easily twice as many eggs as those we observed in the native range. Parallel to *P. canaliculata*, the relationship between estimated volume and EPC implies non-native female *P. maculata* produce larger sized eggs. However, the lower slope of the relationship between mass and EPC suggests non-native clutches contain eggs of greater individual mass than in the native population. Therefore, *P. maculata* females in Texas may have access to higher quality or more abundant food than in their native range. The high access to resources may explain why multiple studies document mean hatching efficiencies from 40% to over 70% for this *P. maculata* population [16,18,20]. Such dramatically larger clutches may represent another example in which, after introduction into a novel environment, characteristics within a non-native population can rapidly differentiate from the source population [34]. Even though individual non-native *P. maculata* clutches contain much higher numbers of eggs, we cannot say at this time if the non-native snails reproduce faster than the native snails without an idea of the average snail's rate of clutch production. Much basic life history information including common parameters such as life span and oviposition frequency remains unpublished for *P. maculata*. 

Although we only collected eggs from one non-native population of each species, we feel our results will allow managers to distinguish between *P. canaliculata* and *P. maculata* in novel populations of both species, as well as across the range of currently established non-native populations. Within their native range, both apple snail species exhibit highly conserved egg size and clutch characteristics across their known distribution [22,23]. Researchers have documented changes in EPC for both apple snail species in response to reduced diet [27]. In such cases, individual egg size and clutch shape remained extremely consistent. Indeed, changes in diet and climate likely account for the differences we observed between the native and non-native populations. We anticipate similar changes in clutch characteristics in other non-native populations in comparable environments, although we need additional research to answer that question. As preliminary support, we can report that a limited number of clutches of *P. maculata* collected from Florida (Loxahatchee National Wildlife Refuge) showed similar morphology and total egg numbers to those collected in Texas (Burks, unpublished data). We expect that our methodology, combined with an understanding of the recent history of apple snail invasion, should still allow correct species identification.

In addition to identifying species based on clutch characteristics, we investigated the most efficient and accurate methods of quantifying apple snail egg clutches. We attempted to exploit the consistent shape of clutches and investigated different mathematical methods of estimating clutch volume from easily measured dimensions. However, a simple model using the product of clutch length and depth with specific functions for each population proved more accurate than more complex models of clutch volume. Using our best model, field crews can survey the reproductive output of an apple snail population by simply measuring clutch length and depth and calculate an approximation of number of eggs per clutch. This approach would save time compared to carefully removing clutches from their substrates and manually counting thousands of eggs. 

Apple snails will oviposit on almost any stable, emergent object, from tree roots to snagged chunks of Styrofoam [12,20]. Future surveys will undoubtedly encounter clutches on a wide range of structures. For our first modeling effort, we chose to use clutches collected from consistent substrates. Fortunately, substrate material (wood versus plastic, plant versus wood) or shape (flat, square, round) has not significantly influenced clutch characteristics in a previous lab study [16]. Furthermore, our best fitting EPC predicting model depends only on clutch length and depth, as opposed to substrate-specific parameters like those in the Intersected Model. Consequently, we remain cautiously optimistic that the functions should extend to clutches oviposited on any substrate. Future studies will be required to provide a consensus.

Due to differences in relationships between clutch characteristics and EPC that we observed comparing native and non-native populations, our population-specific EPC predicting functions may not generalize to all apple snail populations. However, because of the conserved egg size in the native apple snail distribution and the wide range of clutch sizes in our study, we feel the functions for our non-native populations will likely apply to other non-native populations. Therefore, when quantifying egg clutches outside of the populations from which we sampled, we recommend managers utilize either the native or non-native EPC function for their target species, depending on the range of clutch sizes in their area, or follow our methods and develop their own population specific function.

While the Intersected Model proved less accurate than the length and depth models and failed to find an optimal substrate radius for non-native *P. maculata* clutches, our results should not deter researchers from applying similar quantitative methods to other ecological problems. Working through the mathematical processes encouraged us to think critically about patterns in EPC and resource allocation. Furthermore, we found the mathematical results reflected the biological reality in encouraging ways. The model estimated that native apple snails utilized substrates with approximately a 9 mm radius, which corresponds with *Schoenoplectus* species and other thin-stemmed plants utilized by apple snails in their native Uruguay (Burks, personal observations). The model estimated non-native *P. canaliculata* clutches occurred on substrates of 77.0 mm radii and researchers primarily observed non-native *P. canaliculata* clutches on cattails (genus *Typha*) and water lettuce (genus *Pistia*) which have broader, but still rounded, leaves, although that might be somewhat limited to the particular location in Hawaii. Non-native *P. maculata*, however, predominantly oviposit on wild taro (*Colocasia esculenta*) which varies widely in stem radius [20]. The combination of variable substrate radii and wide range of clutch sizes most likely overstretched our data and caused the fitting routines to favor a simpler model. Inclusion of additional data, such as measures of oviposition site radii, would undoubtedly improve the models and we strongly encourage other scientists to explore creative applications of mathematical methods to their research.

## Conclusion

Given the past taxonomic confusion and global distribution of both species [23], the need exists to reliably distinguish between *P. maculata* and *P. canaliculata*. Established populations of NIS quickly negatively impact ecosystems [2,4,24] and early detection represents the first line of defense after an invader enters an area [8]. Ideally, researchers and managers would capitalize on the conspicuousness of apple snail egg clutches in three steps. First, they would inspect emergent vegetation and structures for pink egg clutches to quickly detect the presence of apple snails in a body of water [16]. After spotting eggs, managers would identify the particular species of apple snail by quantifying clutch dimensions and EPC. Finally, the responding agency would assess the egg density and range of the population with field surveys, attempt to locate the source of the invasion, and target control efforts, such as removal of eggs or trapping adults, in the areas containing the highest densities of egg production. Populations of *P. maculata* should receive more rapid attention because of the species’ greater fecundity than *P. canaliculata* [26]. Because the frequency of clutch production remains consistent throughout the reproductive season but EPC can vary widely in response to food availability within an area [27], we strongly recommend managers estimate egg density per area by measuring the dimensions of observed egg clutches and calculating EPC using our models.

 Although this study focused on the two most invasive apple snails in the genus *Pomacea*, the approach of this study applies to other species. Take the recently reclassified species, *Pomacea megastoma* (formerly *Pomella megastoma*), as an example [23]. Much like *P. canaliculata* and *P. maculata*, cryptically-brown adult *P. megastoma* reside mostly beneath the water except when females climb out onto emergent substrata (often rocks) for oviposition of similarly pinkish clutches. Although scientists have not confirmed *P. megastoma* in non-native locations, recent findings regarding the fecundity of this species in its native range (Burks and Plantz, unpublished data) may indicate a high probability of it becoming a pest if introduced to yet uninvaded habitat [26,35]. We found similar clutch shape trends to those observed in the other two apple snail species and future collection and evaluation of clutch characteristic data could distinguish other apple snail species. Furthermore, by adjusting our methods and collaborating with mathematicians, ecologists working with volumes of consistent shapes but variable dimensions could create their own equations to estimate volume based on readily measured dimensions. 

Given the global nature of modern economics and human transit, the number of vectors for non-native invasive species continues to increase [35]. Researchers need to make strides toward stopping the spread of current threats wherever possible because non-native species compete with native organisms, often to the point of causing extensive economic problems, irreversible environmental damage or even extinction [3]. With the results of these investigations, researchers and managers will gain new tools to help quickly and efficiently identify, quantify and control new non-native apple snail populations.
