# Correction: Count Your Eggs Before They Invade: Identifying and Quantifying Egg Clutches of Two Invasive Apple Snail Species (*Pomacea*)

**DOI:** 10.1371/journal.pone.0099149

**Published:** 2014-05-30

**Authors:** The PLOS ONE Staff

There are a number of errors in Table 3. Please see the corrected Table 3 here.

**Table 3:** **Coefficients for best fitting models predicting egg number per clutch**.

Explanatory Variable	Population		Intercept	Slope
Length	*P. canaliculata*	Native	3.75 ± 2.87×10^-2^ (130)	4.30×10^-2^ ± 6.49×10^-3^ (66.3)
		Non-Native	4.31 ± 3.07×10^-2^ (140)	2.41×10^-2^ ± 1.01×10^-3^ (23.8)
	*P. maculata*	Native	5.12 ± 1.83×10^-2^ (280)	3.29×10^-2^ ± 2.95×10^-4^ (112)
		Non-Native	6.65 ± 1.17×10^-2^ (570)	1.62×10^-2^ ± 1.85×10^-4^ (87.5)
Length×Depth	*P. canaliculata*	Native	4.02 ± 2.45×10^-2^ (164)	3.51×10^-3^ ± 5.09×10^-5^ (68.9)
		Non-Native	4.21 ± 2.49×10^-2^ (169)	2.67×10^-3^ ± 7.57×10^-5^ (35.2)
	*P. maculata*	Native	4.88 ± 2.11×10^-2^ (232)	3.49×10^-3^ ± 3.18×10^-5^ (110)
		Non-Native	6.75 ± 9.65×10^-3^ (700)	7.86×10^-4^ ± 8.16×10^-6^ (96.4)
Length×Width×Depth	*P. canaliculata*	Native	4.34 ± 2.01×10^-2^ (215)	1.47×10^-4^ ± 2.11×10^-6^ (70.0)
		Non-Native	4.37 ± 2.02×10^-2^ (216)	1.16×10^-4^ ± 3.18×10^-6^ (36.4)
	*P. maculata*	Native	5.11 ± 1.87×10^-2^ (272)	1.49×10^-4^ ± 1.32×10^-6^ (112)
		Non-Native	6.91 ± 8.32×10^-3^ (830)	2.78×10^-5^ ± 2.99×10^-7^ (93.1)
Mass	*P. canaliculata*	Native	4.12 ± 2.37×10^-2^ (173)	3.77×10^-1^ ± 5.54×10^-3^ (68.0)
		Non-Native	4.24 ± 2.15×10^-2^(197)	4.10×10^-1^ ± 1.01×10^-2^ (40.7)
	*P. maculata*	Native	5.47 ± 1.52×10^-2^ (359)	1.72×10^-1^ ± 1.48×10^-3^ (116)
		Non-Native	6.87 ± 8.48×10^-3^ (810)	8.44×10^-2^ ± 8.63×10^-4^ (97.8)

Coefficients for model with lowest AIC value for each *Pomacea* species (Table 2). Each model is a Generalized Linear Model with a Poisson distribution, such that: 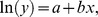
where *y* is egg number per clutch, *a* and *b* represent the intercept and slope coefficients, respectively, and *x* is the explanatory variable. Coefficients include ± one standard error and the corresponding Z-Value in parentheses. The Z-value for each coefficient proved significant (p < 0.0001).
